# Making marine image data FAIR

**DOI:** 10.1038/s41597-022-01491-3

**Published:** 2022-07-15

**Authors:** Timm Schoening, Jennifer M. Durden, Claas Faber, Janine Felden, Karl Heger, Henk-Jan T. Hoving, Rainer Kiko, Kevin Köser, Christopher Krämmer, Tom Kwasnitschka, Klas Ove Möller, David Nakath, Andrea Naß, Tim W. Nattkemper, Autun Purser, Martin Zurowietz

**Affiliations:** 1 GEOMAR Helmholtz Centre for Ocean Research Kiel, Kiel, Germany; 2 National Oceanography Centre, European Way, Southampton, SO14 3ZH UK; 3 MARUM – Center for Marine Environmental Sciences, University of Bremen, Leobener Str. 8, D-28359 Bremen, Germany; 4 Alfred Wegener Institute Helmholtz Centre for Polar and Marine Research, Bremerhaven, Germany; 5 Laboratoire d’Océanographie de Villefranche, Sorbonne Université, 06230 Villefranche-sur-Mer, France; 6 Helmholtz-Zentrum Hereon, Institute of Carbon Cycles, Geesthacht, Germany; 7 DLR/Institute of Planetary Research, Planetary Geology, Berlin, Germany; 8 Biodata Mining Group, Faculty of Technology, Bielefeld University, Bielefeld, Germany

**Keywords:** Ocean sciences, Scientific data

## Abstract

Underwater images are used to explore and monitor ocean habitats, generating huge datasets with unusual data characteristics that preclude traditional data management strategies. Due to the lack of universally adopted data standards, image data collected from the marine environment are increasing in heterogeneity, preventing objective comparison. The extraction of actionable information thus remains challenging, particularly for researchers not directly involved with the image data collection. Standardized formats and procedures are needed to enable sustainable image analysis and processing tools, as are solutions for image publication in long-term repositories to ascertain reuse of data. The FAIR principles (Findable, Accessible, Interoperable, Reusable) provide a framework for such data management goals. We propose the use of image FAIR Digital Objects (iFDOs) and present an infrastructure environment to create and exploit such FAIR digital objects. We show how these iFDOs can be created, validated, managed and stored, and which data associated with imagery should be curated. The goal is to reduce image management overheads while simultaneously creating visibility for image acquisition and publication efforts.

## Introduction

Marine image datasets are critical for monitoring and managing the vast and remote ocean environments in the face of anthropogenic impacts^1–3^. However, a comprehensible image of the global seafloor at a resolution of one millimeter per pixel resolution would require exabytes (millions of terabytes) of data storage^4^. Such a single, static seafloor snapshot would ignore temporal aspects and not even touch on the largest habitat on Earth: the open ocean water column^5^. Repeated imaging for monitoring and the ongoing technical evolution of cameras will increase dataset sizes by many orders of magnitude. Marine research institutions already acquire image data in the petabyte-range annually^6^, and unquantifiable volumes of industry and government data have to be counted in as well^7^. Such enormous dataset sizes present immense challenges for management. The processes of storage, management, linking, and interpretation is carried out by a growing number of national organizations with different financial backgrounds. Each one struggling to find sustainable software solutions for these issues individually, although a much higher degree of cooperation would be required facing the high volume and diversity of data.

Imaging is often conducted to obtain occurrence, abundance, size and taxonomic information on benthic and pelagic fauna^8,9^. Planktonic images can be linked with allometric equations to estimate e.g. respiration rates of zooplankton^10^ and to reconstruct vertical community structure in relation to hydrography^11^. Additionally, particulate matter flux can be estimated from image data at unprecedented spatial and temporal resolution^12,13^. In other use cases, image data can be used for ground-truthing or for making targeted temporal or spatial high-resolution observations of regions of interest^14,15^. This includes both still images (photos) and moving images (videos). Image data can be linked to other data modalities, such as acoustic sensing, through machine-learning techniques^16^ for example. In contrast to satellite-based mapping of the continents (and to some degree this holds also in very shallow water coastal areas), the optical properties of water require that cameras and lights must be brought very close to the image target. Consequently, data are not acquired at a large scale by the same standard satellite configuration, but rather need to be obtained locally by many different parties using diverse, but possibly also intercalibrated imaging systems. Marine image data sets are acquired by a vast diversity of camera systems from various camera platforms, such as AUVs (autonomous underwater vehicles), ROVs (remotely-operated vehicles), towed camera platforms, moorings, Argo floats and submersibles^7^. This has resulted in a high technical heterogeneity of image data sets in terms of resolution, illumination, deployment type, viewing direction, scope, boundary conditions, and target use cases. Examples of published image data sets^17–19^ so far include only a fraction of the required metadata to adhere to all FAIR principles^20^ and thus do not succeed regarding all FAIR metrics^21^. This compounds the question as to which and how different types of image acquisition meta data are recorded with an image set, such as position, orientation, altitude, water properties, etc. Such heterogeneity prevents linking and/or comparison between different image data sets, potentially hindering knowledge discovery and the sustainable use of existing data sets in the future. Therefore, a key objective for the future is to make image data comparable and usable by a wide community of different stakeholders.

Image data are also inherently unstructured when compared to other data types. An image data set cannot be “plotted” like a time series of sensor data, such as a thermometer measurement of temperature at a particular point location. It is thus not easy for humans or machines to extract the semantic content, i.e., the meaning of the things visible in the images, from a data set. This challenges quality control and extraction of information from the image contents. Information extraction from images requires laborious manual inspection and annotation^22^, algorithmic solutions^23^ or both^24,25^. A multitude of tools have been developed in this regard, for example to annotate images or detect important segments of video sequences^26,27^. Yet, no community standard exists to structure, share, or reuse such image-derived information. This gap has been repeatedly identified as part of scientific and environmental management initiatives, such as the UN Ocean Decade program *Challenger 150* (https://challenger150.world), workshops of the UN International Seabed Authority, the *Big Picture* group in the UK or the international Marine Imaging Workshop community (https://www.marine-imaging.com).

All three challenges - data volume, technical heterogeneity and missing semantic structure - can be mitigated. Data has the potential to be distributed between organizations via a federated infrastructure to distribute the storage burden, as well as to facilitate joint analyses approaches using also future science cloud infrastructure services. Standards developed would systematically capture the technical heterogeneity and facilitate links between data sets. Software tools and procedures need to embrace those standards to facilitate efficient information extraction from large or multiple marine image data sets. All of these efforts can be addressed by applying the FAIR principles (Findable, Accessible, Interoperable, Reusable) to marine image data^28^, which represents the current main driver in research data management. In brief, the goal is to i) create visibility for all existing and upcoming data by making them *Findable* via data portals through unique identifiers; ii) enable efficient sharing of and *Access* to data by providing it through standardized interfaces; iii) facilitate links to connected resources for use with the data by providing *Interoperable* links usable by software tools; and iv) allow others to easily *Reuse* data by providing licensing information as well as using standardized data formats^28^.

How to achieve FAIRness for different data types or data use communities is an ongoing research topic^20^. Organizations such as the European Open Science Cloud (EOSC) or the Research Data Alliance (RDA) publish recommendations on FAIRness^29–31^, some of which are directly applicable to marine image data. These include human- and machine-readable standardized vocabularies or the use of persistent identifiers (PIDs). However, the massive data set sizes in marine imaging in monitoring and exploration create additional requirements for FAIRness such as the detachment of the image data from associated data (called metadata) to maintain efficient handling of data and facilitate accessibility and reuse. Finally, the inherent lack of semantic structure in image data, i.e., that information in images is encoded in millions of pixels that only together create a picture by means of interpretation by a brain or computer, requires standards for tools that create and use information derived from images such as annotations.

While substantial previous work has been attempted to address these issues, no (image) metadata standard can address the aforementioned issues completely. Efforts like OceanBestPractices (https://www.oceanbestpractices.org/) are trying to collect standards and methods^32,33^. However, while the bottom-up nature of the loose collection is a valuable step towards reproducibility and comparability, it does not aim at selecting one gold standard to make all data sets interoperable. Established standards such as Schema.org, Dublin Core and Darwin Core provide key terms, yet are too unspecific. Those standards provide the central metadata concepts around which more dedicated standards should be developed. They need to be as generic as they are to accommodate all data types and use cases. Standards from medical imaging such as DICOM (Digital Imaging and Communications in Medicine) or OME (Open Microscopy Environment) are tuned for medical applications and hence incompatible with Earth-science applications^34^. Audubon^35^, MEDIN^36^ and ISO19115 for image data address some aspects required to achieve FAIRness of marine image data. However, they lack components on the purpose of imaging, geometric scaling of image data for size and volume quantification and particularly they lack components to address the content of image data such as derived features or annotations. Hence, those standards are incomplete and too generic to capture the specific demands of imaging in the oceans. The standard that is most compatible to the demands for marine image data is the Planetary Data System (PDS4)^37^. This standard is used by e.g., NASA for remote sensing data acquired by satellites. This standard uses XML to encode metadata and has strong components on data encoding which are relevant for satellite data but not employed in marine imaging which relies on standard data formats for imagery. PDS4 lacks technical aspects to document camera type and setting used for acquisition and is agnostic of imaging use cases. While it may be a good fit for some remote sensing data, it does not cover all requirements of the marine sciences or for marine imaging.

As a solution, we propose the use of image FAIR Digital Objects (iFDOs) and present an infrastructure environment to create and exploit such iFDOs^38^. The iFDO format solves the issues of technical heterogeneity and semantic un-structuredness. The infrastructure environment solves the challenge of handling vast image data sets. But only in combination can the iFDOs and the infrastructure environment that works with iFDOs achieve FAIRness of marine images. iFDOs are designed to be applied to all marine image data: *in-situ* and *ex-situ* imagery, photos and videos and datasets consisting of single images to thousands of images.

## Results

### iFDOs – image fair digital objects

**Fig. 1:**
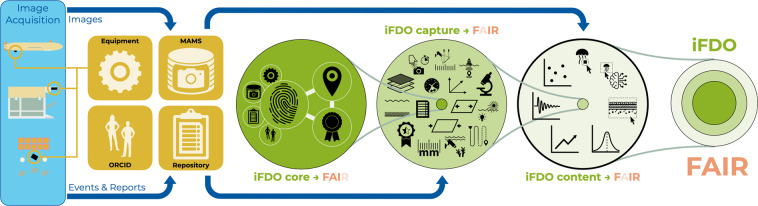
Setup of image FAIR Digital Objects. Key information and image data is stored in a dedicated infrastructure (yellow squares). iFDOs only contain persistent identifiers to those external information resources. Additionally, specific metadata for marine imaging use-cases is stored inside the iFDO files. iFDOs consist of three sections: (1) the required *core* part which includes the persistent identifiers as well as licensing information; (2) the recommended *capture* part that addresses the technical heterogeneity of image acquisition; and the (3) the optional *content* part that captures semantic information from within the images to address the heterogeneous nature of image data. Together, these three sections constitute one iFDO file. This file contains header information on the entire image data set as well as detailed information on each image item within a defined set of images.

iFDOs consist of various metadata fields, grouped into three sections (see Fig. 1). Some fields are required, and some are recommended or optional. FAIRness can only be achieved by populating all the fields in the required iFDO *core* section. Visibility and credit for valuable image data can only be created by populating the recommended fields in the iFDO *capture* section. This section captures how the image data was created. Semantic structure within image data can only be extracted by populating the optional fields in the iFDO *content* section. All three sections of image metadata need to be stored together in one iFDO file. The file must be human and machine-readable, hence the YAML (Yet Another Markup Language) format was chosen for all current tool developments.

See the Methods section of this manuscript or the online material (https://www.marine-imaging.com/fair) for details of the iFDO sections.

### Creating iFDOs

**Fig. 2:**
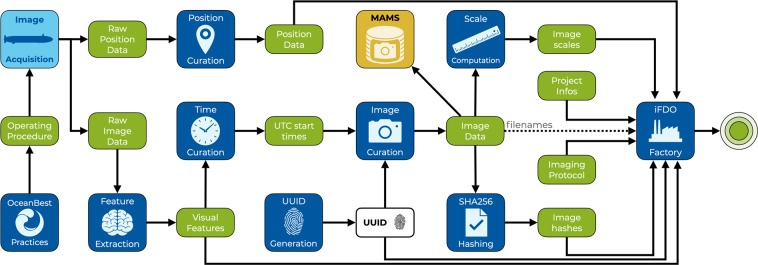
Creating an iFDO. Marine image acquisition is guided by OceanBestPractices and creates raw image data and raw position data (for *in-situ* imaging). Multiple processing steps (blue boxes) create derived data products (green boxes) that are ultimately merged by an iFDO factory process to one iFDO file (green circle).

Creating iFDOs requires a factory process that merges image-derived and acquisition-related information such as position data. This factory process will be a algorithmic method “produces” a digital iFDO object. Figure 2 provides an overview of the most common steps required to create an iFDO. The specific steps for on dataset or use case depend on the type of image acquisition and available accompanying data. In general, it is essential to verify timing of the images in UTC (Coordinated Universal Time) to allow correlation with accompanying data by time point. Position data needs to be curated to remove outliers and smooth tracks. UUIDs (Universally Unique Identifiers) need to be created and handle URLs registered. In an image curation step, UUIDs need to be minted into the image files and images be renamed to include project, timing and camera information. This curated image data can then be made available through FAIR infrastructure and serves as the input to compute further derived information such as image scale (pixels per meter) and to compute SHA256 hashes for file validation. We provide the MarIQT python package (https://gitlab.hzdr.de/datahub/marehub/ag-videosimages/mariqt) to provide algorithms and tools for all these steps. Step-by-step material on iFDO creation is available as an OceanBestPractice document^39^.

### FAIR marine image infrastructure environment

**Fig. 3:**
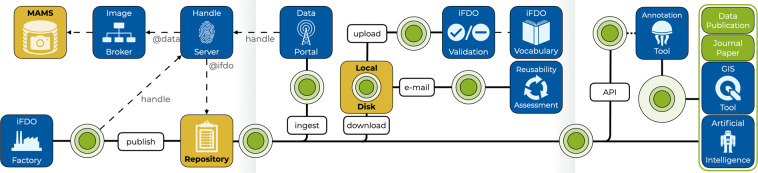
Creation and progression of an iFDO (green circles) and its derived versions. The left part shows how an iFDO uses persistent identifiers to reference itself within the FAIR infrastructure. The middle part shows how iFDO files can be discovered, shared, advertised, and validated. The right part shows how implementing iFDO-compliant APIs to marine science tools facilitates reuse of image data for arbitrary purposes.

iFDOs address only particular aspects of making marine image data FAIR. Additionally, an infrastructure environment is needed that exploits the information contained in iFDOs and that the iFDOs link to through PIDs. Figure 3 shows an overview of building blocks of an appropriate infrastructure environment. Image acquisition is guided by best-practice standard operating procedures (SOPs) and creates data products such as the integration of image data and navigation data, documented by acquisition protocols and accompanied by high-level project information. Acquisition protocols should be published in an open-access data repository such as OSIS (Ocean Science Information System, https://osis.geomar.de/app). Image data should be migrated to a MAMS (Media Asset Management System). This MAMS needs to be a long-term repository by itself or be part of a larger long-term repository infrastructure such as PANGAEA (https://www.pangaea.de) that guarantees long-term availability of image data and image metadata.

An iFDO factory software component collates information from all acquisition products and bundles them into an iFDO file. Persistent identification of images is enabled by assigning UUIDs. UUIDs are generated at random for each image item, with an additional UUID generated for the entire image set. These UUIDs constitute a key component that addresses several FAIR metrics and should be a part of the handle URLs, linking to the image data and metadata. The initial iFDO file needs to be published in an open access data repository such as OSIS (Ocean Science Information System), PANGAEA or another long-term repository featuring persistent identification. A handle server is required as a single-point-of contact to resolve requests for data. Such a handle server can cover use cases beyond image data. This is the key building block to facilitate accessibility of both the image data and image metadata. DOI systems are implicitly included here as the DOI system is based on handles.

Once an iFDO file has been published in a long-term repository, it can be viewed, downloaded, and shared. A user can download an iFDO file to a local disc and continue working with it. Sharing of iFDO files via e-mail is possible as the file size rarely exceeds a few hundred kilobytes and larger files can be easily compressed. With information from the iFDO core and capture sections it is possible to assess the reuse potential of an image data set (https://www.marine-imaging.com/selection.html). All holders of iFDO files possess the link information needed to access the image data or updated versions of the iFDOs in the form of handle URLs. By ingesting the information from an iFDO into a data portal (e.g., https://marine-data.de, https://emodnet.ec.europa.eu/en/portals) this data set is made visible and thus Findable by the global community. Discoverability of image data by machines or humans should further be improved by establishing catalogue services, e.g., using CSW (Catalogue Service for the Web), through those portals or as linked infrastructure.

The key building block for Interoperability is the adherence to a controlled iFDO vocabulary. This vocabulary is defined by the iFDO core, capture and content fields. Validation of an iFDO file against the vocabulary corresponding to the version of a particular iFDO is a required tool for quality control.

Incentives are required to establish iFDOs as the exchange format for image metadata and to promote an exhaustive usage of its features. Such incentives are created by providing established community tools with plugins or API (application programming interface) extensions that allow using iFDOs in particular tools. At present, integration plugins into the Robot Operating System (ROS) for marine robotics, an integration into BIIGLE for image and video annotation and an integration into QGIS for geographical mapping are under development. These tools will be released in 2022. Once a growing group of tools can exploit information from iFDO files, Reusability of image data products will be achieved.

Aside from the SOPs for image acquisition, SOPs on iFDO creation and image curation were developed by the MareHub working group^39,40^. These SOPs complement this manuscript by e.g., providing hands-on material for expeditions.

### FAIRness of marine images

With the iFDO concept implemented and the required infrastructure environment in place, most recommendations on FAIRness can be addressed^29,30^. Table 1 provides an overview on the status for marine images using iFDOs and the infrastructure environment. Of the 41 RDA recommendations, 36 are achieved, 2 are not viable in the image data context and 3 are in preparation for upcoming versions of the iFDO standard. All essential and important FAIR indicators are being addressed by iFDOs and the marine image infrastructure environment.

**Table 1:** Overview of FAIR recommendations and their implementation for marine images.

Recommendation	Description	Implementation
RDA-F1-01M*	*Metadata is identified by a persistent identifier*	URL:handle@ifdo
RDA-F1-01D*	*Data is identified by a persistent identifier*	URL:handle@data
RDA-F1-02M*	*Metadata is identified by a globally unique identifier*	image-set-uuid
RDA-F1-02D*	*Data is identified by a globally unique identifier*	image-set-uuid
RDA-F2-01M*	*Rich metadata is provided to allow discovery*	iFDO format
RDA-F3-01M*	*Metadata includes the identifier for the data*	URL:handle
RDA-F4-01M*	*Metadata is offered in a way to be harvested and indexed*	iFDO by https
RDA-A1-01M^+^	*Metadata contains info to enable accessing the data*	URL:handle@data
RDA-A1-02M*	*Metadata can be accessed manually*	URL:handle
RDA-A1-02D*	*Data can be accessed manually*	URL:MAMS
RDA-A1-03M*	*Metadata identifier resolves to a metadata record*	URL:handle@ifdo
RDA-A1-03D*	*Data identifier resolves to a digital object*	URL:handle@data
RDA-A1-04M*	*Metadata is accessed through standardized protocol*	https
RDA-A1-04D*	*Data is accessible through standardized protocol*	https, nfs, …
RDA-A1-05D^+^	*Data can be accessed automatically*	MAMS-API
RDA-A1.1-01 M*	*Metadata is accessible through a free protocol*	https
RDA-A1.1-01D^+^	*Data is accessible through a free access protocol*	https
RDA-A1.2-01D	*Data is accessible with authentication and authorization*	https
RDA-A2-01M*	*Metadata is guaranteed to remain available after data loss*	handle system
RDA-I1-01M^+^	*Metadata uses knowledge representation in standard format*	iFDO format
RDA-I1-01D^+^	*Data uses knowledge representation in standard format*	e.g. jpg, png, mov
RDA-I1-02M^+^	*Metadata uses machine-understandable knowledge representation*	yaml
RDA-I1-02D^+^	*Data uses machine-understandable knowledge representation*	e.g. jpg, png, mov
RDA-I2-01M^+^	*Metadata uses FAIR-compliant vocabularies*	in preparation
RDA-I2-01D	*Data uses FAIR-compliant vocabularies*	in preparation
RDA-I3-01M^+^	*Metadata includes references to other metadata*	ORCID etc.
RDA-I3-01D	*Data includes references to other data*	image-uuid
RDA-I3-02M	*Metadata includes references to other data*	image-set-uuid
RDA-I3-02D	*Data includes qualified references to other data*	N/A for images
RDA-I3-04M	*Metadata includes qualified references to other data*	N/A for images
RDA-I3-03M^+^	*Metadata includes qualified references to other metadata*	iFDO v2.0.0
RDA-R1-01M*	*Plurality of accurate and relevant attributes are provided for reuse*	iFDO capture & content fields
RDA-R1.1-01 M*	*Metadata includes information about the reuse license*	iFDO core fields
RDA-R1.1-02 M^+^	*Metadata refers to a standard reuse license*	iFDO core fields
RDA-R1.1-03 M^+^	*Metadata refers to a machine-understandable reuse license*	iFDO v2.0.0
RDA-R1.2-02 M	*Metadata includes cross-community provenance info*	iFDO v2.0.0
RDA-R1.2-01 M^+^	*Metadata includes community-specific provenance info*	iFDO capture & content fields
RDA-R1.3-01 M*	*Metadata complies with a community standard*	iFDO file format
RDA-R1.3-01D*	*Data complies with a community standard*	e.g. jpg, png, mov
RDA-R1.3-03 M*	*Metadata uses a machine-understandable standard*	yaml
RDA-R1.3-02D	*Data uses a machine-understandable standard*	e.g. jpg, png, mov

Recommendations are ranked by the RDA as “Essential” (*), “Important” (+) and “Useful”.

## Discussion

Turning the FAIR principles into reality for different data types is a process^38^, so achieving FAIRness will remain a challenge. Even with all required infrastructure in place, the task to keep data sets FAIR will continue in perpetuity. The presented iFDO standard and the infrastructure environment represent a large step towards achieving FAIRness for marine image data, particularly conceptually. Neither standardization nor infrastructure provision alone is the solution, though, and both aspects require considerable resources for setup, operation, quality control, and management into the future.

iFDOs follow recommendations by the EOSC and RDA and are built upon existing generic standards^29,30^. iFDOs are compatible with those standards and key terms can be mapped directly to those standards. By being extendable to further marine domain-specific terms, iFDOs remain flexible. Therefore an evolution of their vocabulary is expected, in particular given the ongoing technological changes associated with marine imagery and the variability in marine imaging use in these times of increasing pressures on the global marine environment such as global warming and marine resource exploitation.

The infrastructure building blocks to curate marine imagery naturally differ between institutes, sectors, or nations. By adhering to a common interface – such as the iFDO format – differences in infrastructure, or changes over time become irrelevant as the overall environment remains functional. Yet, keeping infrastructure operational for long-term use requires sufficient funding. Without such infrastructure, such as long-term repositories, FAIRness for images cannot be achieved.

By also providing specific implementations for components of the infrastructure environment, we support adoption of iFDOs in existing workflows. We currently develop software to create iFDOs, after cruises or already during marine robotic deployments, as well as plugins to established tools such as BIIGLE^41,42^ or QGIS. Furthermore, we develop vocabulary servers to validate iFDO versions and tools to assess the reusability of data sets for different use cases.

To address all current requirements of FAIRness (see Table 1), additional building blocks are needed. These include setting up a machine-readable vocabulary server. On top of that an iFDO validation service is needed, in addition to the validation algorithms already existing in the MarIQT Python package (https://gitlab.hzdr.de/datahub/marehub/ag-videosimages/mariqt). Qualified references to other metadata need to be implemented in the iFDOs. For example, the ORCID of an image-pi is already a part of the iFDO but this remains just a number, not a URL to resolve it. The same applies to the machine-readability of the reuse license. To achieve legal bindingness of the linked license an additional Handle URL (or location of a handle) may have to be added, linked directly to the full text of a license.

At present, the iFDO concept is designed for and applied to raw and processed image items. The concept is flexible enough to also accommodate for derived data products such as 3D reconstructions, semantic maps or full depth-of-field sharpness reconstructions. To make such image-derived data products FAIR requires incorporation of provenance information^43^. This is the final missing aspect to address all FAIR recommendations for images. However, the topic is still under discussion in the data management community, without an obvious and modern solution being presented. Hence addressing provenance will likely remain an open task for later iFDO versions.

Using YAML as the file format was a community choice led by practical considerations on human- and machine-readability as well as compactness. In practice, XML or JSON format would be alternatives, but were dismissed due to their larger file size, inferior readability and due to their relative complexity resulting in an unnecessary entry barrier for adopters of the iFDO standard. At present, iFDO files rarely exceed a few kilobytes in size. A potential development in the future might allow compression of iFDO files for even lower file sizes.

An adoption of the principles by the community is needed to truly call the iFDO format a *standard* and the workflows within the infrastructure environment *standard* operating procedures. This requires the development of a common data culture for marine imaging^44^. The framework presented here, to incorporate FAIR principles for marine images, aims to provide a basis for discussion to develop that culture. Benefits include the potential for Big Data scientific analyses, reducing effort in management, enabling Interoperability and simplification of tools and their development, increased data credibility, and an increase in the visibility of and credit for imaging efforts. This is an investment into the future that we believe to be worthwhile. Reduced effort is first provided through tools that mask the complexity of iFDOs whilst preserving their full potential. Secondly, effort in image analysis workflows is reduced as tools become interoperable.

Once the ideal of FAIRness of marine images has been achieved, then data discovery, sharing, analysis and publication will run smoothly with all the complexity and tediousness of metadata hidden from the users. This will benefit scientific and blue economy uses of marine image data and enable a data capacity transfer. iFDOs and the FAIR infrastructure environment represent a step towards that future.

Overall, our goal is to establish iFDOs as the key data standard for marine image analysis in diverse infrastructure environments. Once a critical mass of compatible tools and users is reached, the complexity of iFDOs and the infrastructure environment are envisioned to disappear. This is because the standardized format and standardized interfaces will make working with marine images more efficient and more effective. Adoption will yield streamlined research, with more robust data foundation and facilitate truly open and interdisciplinary use of marine image data. This should overall be beneficial for ocean knowledge discovery, protection, and sustainable use of the oceans, thus addressing directly certain specific development goals set by the UN. FAIR imagery contributes to the generation of robust essential ocean variables^45^.

Using these principles and tools will benefit scientists as well as governance institutes, provide transparency in scientific data collection and thus will help keeping the general public informed. It will allow funding agencies to quantify image-related project outcomes and reduce the costs of data usage by increasing the reuse potential. This is of particular interest with regard to machine-learning applications where standardized image metadata and standardized image annotations are needed. Here, iFDOs can foster efficient reuse and validation by evolved machine-learning systems and further support making image annotations reusable and comparable through providing a standardized data format.

We, the authors, commit to embracing the potential of iFDOs. We will strongly encourage and support software development for their adoption in key community tools and we have begun doing so. We will develop tools to enrich our upcoming data sets with iFDOs and support initiatives to enrich existing data sets with iFDOs. We strongly believe in FAIR marine image data and invite contributions to drive the evolution of the concept and its implementation.

## Methods

This manuscript always uses the term *images* according to the Dublin Core definition throughout. Images refers to both still images (photos) as well as moving images (videos). This manuscript is not a handbook or tutorial on all aspects of iFDOs and the FAIR marine image data infrastructure environment. The online resources (https://www.marine-imaging.com/fair) contain more complete and hands-on material.

### iFDO parts

An iFDO file consists of two parts: the image-set-header part and the image-set-items part. The header part contains default values for all items. The image-items part contains all values that deviate from the default values for this specific image item. They supersede the default values. All metadata of an image item is provided in a list. For moving images, the first entry of the list contains an object of those metadata fields that are defaults for an entire video. All subsequent list entries correspond to specifications of the metadata for one given timepoint of a video. For still images this list may contain only one entry of its field values or it is rather a single object of field values for this particular photo.

### UUIDs

Making data FAIR requires that data is assigned a persistent identifier (PID). Many such PID systems exist, and usually they are based on the handle system (e.g., DOI, ORCID) and alpha-numerical IDs that are globally unique. For iFDOs, we chose to use UUIDs (Universally Unique Identifiers), more precisely the random UUID type 4. These UUIDs can be created by anyone. There are ca. 5 × 10^36 possible UUID4s, making it almost impossible that the same UUID is created more than once. This UUID is the alphanumerical identifier that must be assigned to each image and to each image set. The UUID for the image file (a photo or video) must be written into the metadata header of the image file itself to be included into the file hash later (see below). How this step can be done depends on the image file format used. Mostly, software tools like *exiftool* or *ffmpeg* are used which is also the case in the MarIQT Python package. The image-set-uuid only needs to be part of the iFDO file and not written to any image file.

Using UUIDs for persistent identification is essential to achieve FAIRness by making images uniquely identifiable. Those UUIDs need to be registered in a long-term data repository and data portal to facilitate Findability of images.

### File hashes

Hashes are used in iFDOs to monitor the integrity of images. A hash is a fingerprint of a file, computed from the file’s byte content. A file hash can be used to assert that a file is not broken or that a particular file is a specific version. Checking the integrity of a file with hashes requires that the byte content does not change. It is therefore essential, that the UUID is written to the image file’s metadata header *before* the hash for that file is computed!

### iFDO core section - required

An entire image set is defined by project-specific metadata (e.g., deployment, station, dive, mission) and requires information on the ownership and allowed usage of the collection. Numerical metadata is required for each image do document its acquisition position.

The image-set-header part of an iFDO must contain three fields that must not be superseded by values in the image-set-items part. These are image-set-name,image-set-uuid and image-set-handle. All other fields may be provided as default values in the image-set-header, potentially superseded by values in the image-set-items part. These correspond to imaging context (image-context,image-project,image-event,image-platform, image-sensor, image-abstract), the navigation data (image-datetime,image-latitude, image-longitude,image-depth,image-coordinate-reference-system, image-coordinate-uncertainty-meters), image identification (image-uuid, image-hash-sha256) and the reuse permissions (image-pi,image-creators, image-license,image-copyright).

### iFDO capture section – recommended

Information on how image data was captured can be crucial to understand the information subsequently extracted from images. It is thus highly recommended to enrich all iFDOs with *capture* information. This section is expected to grow with time, as additional (marine) imaging domains make use of the iFDO concept and extend the standard with fields for their specific use case.

Existing iFDO *capture* fields currently fall into four categories. First, iFDO *capture* fields with restricted values that characterize the image material (e.g., image-acquisition: photo, video, slide; image-deployment: mapping,stationary,survey, …; image-illumination: sunlight,artificiallight, mixed light;image-capture-mode: timer, manual, mixed; etc.). Second, numerical information on image capture (e.g., image-area-square-meter, image-meters-above-ground,image-overlap-fraction, etc.). Third, pose and calibration information (e.g., image-camera-housing-viewport, image-camera-calibration-model, etc.). Fourth, information on the scope and limitations of the image acquisition (e.g., image-objective,image-target-environment,image-fauna-attraction, image-temporal-constraints, etc.).

Not all iFDO capture fields have to be populated for each data set. But by providing a more complete set of field values for a data set is expected to increase its value and reuse potential significantly, in particular for yet unknown uses. Investing into the iFDO *capture* section will generate the visibility and credit for imaging efforts. By limiting some fields to restricted values, it is possible to classify and filter image data sets in data portals and to rapidly visualize data characteristics. See the *iFDO fixed term icons* section below for details.

### iFDO content section – optional

The iFDOs *content* section fields are a mechanism to encode the semantic content of image data. Some fields encode quantitative scalar data extracted from the image material (e.g., image-entropy,image-particle-count,image-average-colour). Other fields encode higher-dimensional feature descriptors (e.g., image-mpeg7-colorstructure).

Most relevant for marine science is the iFDO *content* section regarding annotations. These are semantic classifications of groups of pixels in the images assigned by humans or algorithms. By establishing iFDOs as the standard format for image annotations, exchange and reuse is facilitated of this derived data while in parallel also making annotation data FAIR. Annotations require a set of semantic labels in the image-annotation-labels field and a set of identifiers of humans or machines in the image-annotation-creators field. The image annotations are then encoded in the image-annotations field as a list of objects. These objects consist of a set of pixel coordinates, one or many label IDs, one or many annotator IDs and an optional confidence value. The format of the pixel coordinates is flexible such that point annotations, bounding boxes, polygons or whole image annotations can be stored.

### iFDO fixed term icons

Some iFDO fields may only be set to a restricted set of values. These restricted fields can be used to group or filter data sets. It is further possible to encode the allowed values with icons, to enable a rapid overview by humans on the content of an image data set. Such icons can be used in documents like data processing reports to provide a quick visual access to a dataset. The complete set of icons can be found in the online material (https://www.marine-imaging.com/fair). The icons are released for public use under CC-0.

### Developing the iFDO format and setting up the infrastructure environment

This work originates in discussions of the Marine Imaging Community workshops^7,46^ and the MareHub initiative of the Helmholtz association. It builds upon concepts that have since evolved into the presented principle^18^. Draft versions of iFDOs were developed in cross-institute and international working groups. Feedback from three stakeholder groups was incorporated into the current version of the iFDO format and documentation: data managers, researchers, and research software engineers. The exchange platform is an open Gitlab group (https://gitlab.hzdr.de/datahub/marehub/ag-videosimages). It contains the complete iFDO documentation, Python code and Jupyter notebooks and is used to receive stakeholder feedback and contributions in the form of tickets and merge requests. The status of the iFDO format and documentation FAIR marine imaging are published through the Marine Imaging Workshop community websites (https://www.marine-imaging.com/fair).

Further developments of the iFDO concept will rely on its adoption by the community. Evolution of the format is expected and welcomed. Future versions of iFDOs may include many other fields that at present were not anticipated. By allowing for arbitrary metadata content within the iFDO files, this is already possible. The core set of mandatory fields however may not change, as those fields are essential to achieving FAIRness of marine image data. They may be extended in the future to include upcoming data management concepts such as provenance information.

## Data Availability

An example iFDO is available at: https://hdl.handle.net/20.500.12085/9a1f8a29-2552-4c11-895b-051f7424d2d2@ifdo.

## Code availability

The Python library MarIQT is available as open source and under rapid development. Contributions in the form of issue submissions and merge requests are welcome. https://gitlab.hzdr.de/datahub/marehub/ag-videosimages/mariqt.
