# Publisher Correction: Making marine image data FAIR

**DOI:** 10.1038/s41597-022-01567-0

**Published:** 2022-07-26

**Authors:** Timm Schoening, Jennifer M. Durden, Claas Faber, Janine Felden, Karl Heger, Henk-Jan T. Hoving, Rainer Kiko, Kevin Köser, Christopher Krämmer, Tom Kwasnitschka, Klas Ove Möller, David Nakath, Andrea Naß, Tim W. Nattkemper, Autun Purser, Martin Zurowietz

**Affiliations:** 1 GEOMAR Helmholtz Centre for Ocean Research Kiel, Kiel, Germany; 2 National Oceanography Centre, European Way, Southampton, SO14 3ZH UK; 3 MARUM – Center for Marine Environmental Sciences, University of Bremen, Leobener Str. 8, D-28359 Bremen, Germany; 4 Alfred Wegener Institute Helmholtz Centre for Polar and Marine Research, Bremerhaven, Germany; 5 Laboratoire d’Océanographie de Villefranche, Sorbonne Université, 06230 Villefranche-sur-Mer, France; 6 Helmholtz-Zentrum Hereon, Institute of Carbon Cycles, Geesthacht, Germany; 7 DLR/Institute of Planetary Research, Planetary Geology, Berlin, Germany; 8 Biodata Mining Group, Faculty of Technology, Bielefeld University, Bielefeld, Germany

**Keywords:** Ocean sciences, Scientific data, Ocean sciences, Scientific data

Correction to: *Scientific Data* 10.1038/s41597-022-01491-3, published online 15 July 2022

In the html version of this article the affiliation details for Rainer Kiko were incorrectly given as ‘Alfred Wegener Institute Helmholtz Centre for Polar and Marine Research, Bremerhaven, Germany’ but should have been ‘Laboratoire d’Océanographie de Villefranche, Sorbonne Université, 06230, Villefranche-sur-Mer, France’ and the affiliation details for Janine Felden, Christopher Krämmer and Autun Purser were incorrectly given as ‘Laboratoire d’Océanographie de Villefranche, Sorbonne Université, 06230, Villefranche-sur-Mer, France’ but should have been ‘Alfred Wegener Institute Helmholtz Centre for Polar and Marine Research, Bremerhaven, Germany’. This has now been corrected in the HTML version of the Article. The PDF version of the Article was correct at the time of publication.

